# Quality assessment of colour fundus and fluorescein angiography images using deep learning

**DOI:** 10.1136/bjo-2022-321963

**Published:** 2022-11-23

**Authors:** Michael König, Philipp Seeböck, Bianca S Gerendas, Georgios Mylonas, Rudolf Winklhofer, Ioanna Dimakopoulou, Ursula Margarethe Schmidt-Erfurth

**Affiliations:** Department of Ophthalmology and Optometry, Medical University of Vienna, Wien, Austria

**Keywords:** Imaging

## Abstract

**Background/aims:**

Image quality assessment (IQA) is crucial for both reading centres in clinical studies and routine practice, as only adequate quality allows clinicians to correctly identify diseases and treat patients accordingly. Here we aim to develop a neural network for automated real-time IQA in colour fundus (CF) and fluorescein angiography (FA) images.

**Methods:**

Training and evaluation of two neural networks were conducted using 2272 CF and 2492 FA images, with binary labels in four (contrast, focus, illumination, shadow and reflection) and three (contrast, focus, noise) modality specific categories plus an overall quality ranking. Performance was compared with a second human grader, evaluated on an external public dataset and in a clinical trial use-case.

**Results:**

The networks achieved a F1-score/area under the receiving operator characteristic/precision recall curve of 0.907/0.963/0.966 for CF and 0.822/0.918/0.889 for FA in overall quality prediction with similar results in most categories. A clear relation between model uncertainty and prediction error was observed. In the clinical trial use-case evaluation, the networks achieved an accuracy of 0.930 for CF and 0.895 for FA.

**Conclusion:**

The presented method allows automated IQA in real time, demonstrating human-level performance for CF as well as FA. Such models can help to overcome the problem of human intergrader and intragrader variability by providing objective and reproducible IQA results. It has particular relevance for real-time feedback in multicentre clinical studies, when images are uploaded to central reading centre portals. Moreover, automated IQA as preprocessing step can support integrating automated approaches into clinical practice.

**WHAT IS ALREADY KNOWN ON THIS TOPIC**

- Current automated approaches for image quality assessment show good performance in binary classification, but typically lack to provide detailed feedback or reasoning for model predictions in most cases.

**WHAT THIS STUDY ADDS**

- By predicting the image quality in multiple categories together with uncertainty for each prediction, we introduce an additional level of detail and promote model interpretability in terms of explainable artificial intelligence (AI). Furthermore, we propose a method for predicting the quality of entire visits, showing promising results towards use in clinical routine.

**HOW THIS STUDY MIGHT AFFECT RESEARCH, PRACTICE OR POLICY**

- The presented automated approach gives clinicians or device operators the opportunity to react to poor quality images in real time, helping to ensure a certain level of image quality and therefore quality of interpretation by clinicians. Moreover, the AI model can be applied as a crucial preprocessing step for other automated image-based approaches, helping to integrate automated approaches into clinical practice.

## Introduction

Good image quality depicts an important aspect for imaging services within reading centres and daily clinical practice, as ophthalmologists routinely use imaging data to assess diseases, disease progression and decide on treatment for patients.^1^ Imaging data such as colour fundus (CF) photographs or fluorescein angiography (FA) are used for analysing retinal morphology, including retinal vasculature, optic disc or presence of pathology.^2^ At the same time, interpretability can be affected or even impossible due to poor contrast, focus or modality specific artefacts including illumination or shadow and reflection for CF and noise for FA.^2 3^ Therefore, image quality assessment (IQA) is an important preceding step to ensure that diagnosis, patient management and treatment decisions are not delayed, hindered or decreased in quality.^2 3^

However, the process of IQA can be resource intensive and time consuming due to the growing amount of data. Manual IQA suffers from intragrader and intergrader variability and is not feasible for most tasks, especially those with urgency. Automated approaches can help to overcome this limitation: With the correct setup and integration of algorithms into the workflow, costs can be reduced drastically, for example, human resources and response time.^2 4^ Furthermore, quality metrics based on quantitative analysis can ensure objective and reproducible results independent from human subjectiveness.

Automated IQA enables real-time feedback on image quality directly after acquisition, allowing to adjust or repeat the imaging process immediately in case of poor quality, ideally while the patient is still on-site. This saves time and reduces burden for both the examiner and the patient, avoiding unnecessary appointments. This applies for clinical practice as well as multicentre study settings, where images are uploaded to a central reading centre.

Another field of application beyond the manual analysis by clinicians is the preprocessing of images for artificial intelligence (AI) analyses. When applied to poor quality images, AI algorithms are often unstable or completely fail to produce meaningful predictions. Assuring adequate image quality for further processing ensures proper functionality of the model.

**Figure 1:**
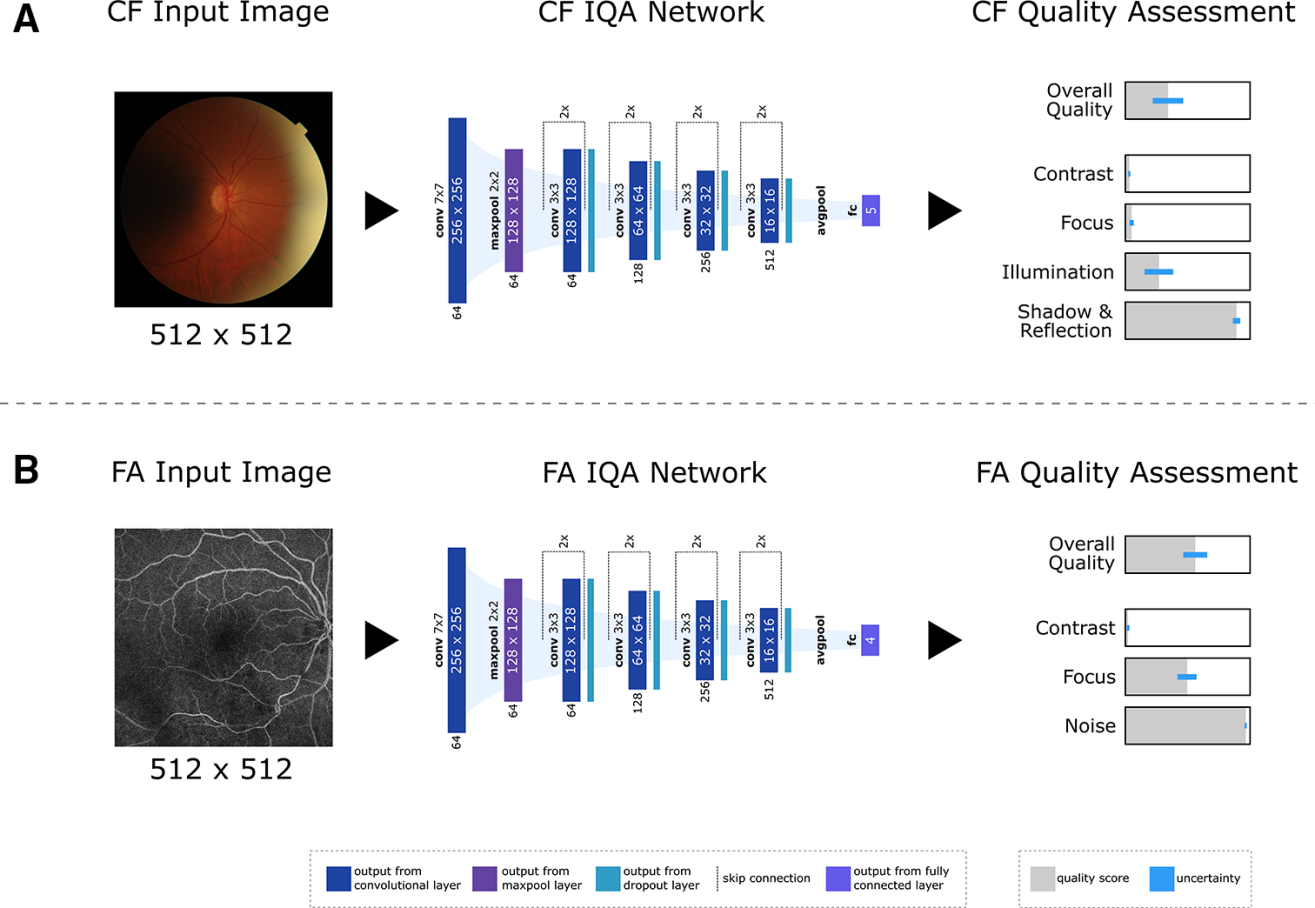
Overview of the proposed image quality assessment (IQA) approach. An input (A) colour fundus (CF) or (B) fluorescein angiography (FA) image is processed by a deep neural network and outputs a quality score for each target category, representing the probability of good quality (depicted in grey). A quality score of 1 relates to worst possible quality, represented as full grey bar. In addition, the model also provides uncertainty scores for each prediction, indicating how confident the model is about its quality score (illustrated in blue on the right-hand side).

In this study, we developed an AI-based approach for fully automated quality assessment of CF and FA images, predicting four (contrast, focus, illumination, shadow & reflection) and three (contrast, focus, noise) modality specific image quality categories for each input image (figure 1). In addition, the models provide an uncertainty score for each prediction, allowing better interpretability of the model output. Beyond a quantitative and qualitative evaluation on a heterogeneous dataset, external dataset and human grading, we also provide a clinical trial use-case evaluation on complete image series of patient visits.

## Materials and methods

### Image datasets

Images and manual annotations provided by the Vienna Reading Center (VRC) from large prospective multicentre trials were used forming two different datasets, one for CF and one for FA. The datasets cover a variety of diseases, including age-related macular degeneration, diabetic macular oedema and diabetic retinopathy. The images have been acquired by more than 200 clinical sites and different manufacturers. Varying acquisition modes result in heterogeneous field of views. Per standardised protocol of the VRC, the field of view is always 30°–60° and always 3–16 images per eye that just show fields that are relevant for each disease. Pixel resolutions range from 496×512 to 6000 × 4000 pixels.

We used two different types of manual annotations: first, one senior grader (highest of five levels) generated binary annotations, classifying images for usage within clinical trials as good/poor quality within each category. These labels were used for training the AI model together with samples from a previous work,^5^ annotated in the same categories by the same graders without a label on overall quality. The second type of label was used for evaluation. A retina specialist, who is also an experienced image grading supervisor at the VRC, assessed the image quality for each category using a Likert scale from 1 (best) to 5 (worst). This fine-grained scale was both employed for validation and test set, allowing a more detailed assessment of the AI model, provided in the online supplemental file 1.

10.1136/bjo-2022-321963.supp1 Supplementary data

This results in two datasets: ‘CF quality’ consists of 2272 CF images from 281 visits from 248 patients. Each image was assessed in the categories ‘contrast’, ‘focus’, ‘illumination’, ‘shadow & reflection’, while 81% of all images were assessed in ‘overall quality’, with a respective share of 0.37/0.59/0.21/0.61/0.38 poor quality labels (0.43 in average). The second dataset ‘FA quality’ comprises 2492 images from 511 visits from 457 patients. FA annotations contain labels for the categories ‘contrast’, ‘focus’, ‘noise’ and for 74% of all images ‘overall quality’, with a share of 0.43/0.62/0.27/0.56 (0.47 in average).

To evaluate inter-reader variability and provide an additional comparison for the proposed AI model, a second senior level grader manually annotated the image quality using the aforementioned Likert scale on the test set.

For external validation of the CF model, we use the public Eye-Quality (EyeQ) Assessment Dataset,^6^ which provides image quality labels ‘Good’, ‘Usable’ and ‘Reject’.

For the additional clinical trial use-case evaluation, the two datasets ‘CF visit quality’ and ‘FA visit quality’ were used. For a subset of the ‘CF quality’ and ‘FA quality’ validation/test sets, the single images are extended through full image stacks of the respective visit. For both datasets, each visit was manually annotated by graders of different levels assessing the overall image quality of the entire visit with a binary label of good/poor quality. This highly subjective grading can depend on the visual perceptibility of a various number of images and their relevance for conducting the corresponding underlying study from a clinical point of view. To create a balanced dataset, all good quality visits and the same amount of randomly selected poor quality samples have been used. This resulted in 1206/66/65 and 3116/106/104 images/visits/patients for CF and FA, respectively, following the same validation/test split as the ‘CF quality’ and ‘FA quality’ datasets. Additional dataset details are included in the online supplemental file 1.

### Technical setup: deep learning method

The developed AI approach predicts the probability of good/poor quality of CF or FA images for multiple categories in addition to the ‘overall quality’, allowing a better reasoning and interpretability for the human operator. Moreover, the model provides an uncertainty score for its prediction, using Monte Carlo Dropout.^7^ Here, dropout layers stay activated for the evaluation. Inference is conducted 16 times for a single sample, using the average of the predicted probabilities as final probability score and the variance as corresponding uncertainty estimate. It is important to emphasise that this uncertainty indicates the confidence of the model regarding its predicted probability score, indicating to which extent we can trust the prediction of the model, and not the likelihood of good/poor quality. An overview of the presented method is shown in figure 1.

The structure of the convolutional neural network (CNN) follows a ResNet-18 architecture,^8^ with additional dropout layers in each block. We use a transfer learning strategy, pretraining the network on the natural image database ImageNet.^9^ Details of the architecture and training are provided in the online supplemental file 1.

### Experimental set-up

Both datasets (‘CF quality’, ‘FA quality’) were split into a training, validation and test set on a patient level, the same patient occurring only in one data subset. The training sets consist of data samples with binary labels. For samples without a label on ‘overall quality’, no loss was calculated for this category and only model weights for the remaining categories were adapted. The data with more fine-grained annotations were randomly split into validation and test set with an approximate ratio of 1:2. This resulted in a train/validation/test split of 1922/87/263 images from 89/40/119 patients for ‘CF quality’ and 2055/116/321 images from 70/100/287 patients for ‘FA quality’.

The training set was used for training the model, while hyper-parameter and model selection were based on the validation performance. The test set was used to evaluate the final performance of the model. For comparison, we evaluated the annotations of the second grader, using the annotations of the first reader as ground truth.

A comparison of the presented AI method with a handcrafted feature-based machine learning approach^5^ based on Pires Dias *et al* 10 is provided in the online supplemental file 1.

### Metrics and evaluation

For each category, we computed accuracy, precision, recall, F1-score, area under the receiving operator characteristic curve (AUC-ROC) and precision recall curve (AUC-PRC). To enable quantitative evaluation with binary model predictions, the Likert scale annotations on the validation and test set were mapped to binary labels (1–2: good image quality, 3–5: poor image quality). Details on the used evaluation metrics and the manual label distribution per category are provided in the online supplemental file 1. Regarding the evaluation on the EyeQ dataset,^6^ we used the provided training set for selecting the optimal threshold of the prediction probability, while evaluation was conducted on the test set. McNemar’s test with ‘alpha’=0.05 was used to test for statistical significant differences.

#### Clinical trial use-case evaluation—visit quality

While previously published image quality detection methods on CF and FA were trained and evaluated on single images, within a clinical trial whole image series are typically acquired during a single patient visit. Clinicians are therefore confronted with the task of judging the overall quality of an entire image series.

To be able to predict the quality of entire visits, the image level predictions are combined into a visit level score by averaging the binary image level predictions, which again results in a score between 0 and 1. A detailed description of this process is provided in the online supplemental file 1.

In this experiment, we evaluate the performance of the AI model on this clinical trial use case, comparing the network predictions with the human visit level labels on the ‘CF visit quality’ and ‘FA visit quality’ test sets.

## Results

### Quantitative results

An overview of the quantitative results per modality and category is provided in table 1. We observed a similar performance behaviour in both modalities throughout different categories. In particular, the developed networks achieve best performance in the task of classifying the ‘overall quality’ with a F1-score/AUC-ROC/AUC-PRC of 0.907/0.963/0.966 for CF and 0.822/0.918/0.889 for FA. The best performance for specific categories was achieved for ‘*focus’* and ‘*contrast’*, while lowest scores were obtained in modality specific categories: ‘*illumination’* and *‘shadow & reflection’* in CF and ‘*noise’* in FA. These are also the categories with biggest label imbalance within training data. In all categories, the DL approach achieves human-level performance or even significantly outperforms the human grader (table 1).

**Table 1:** Results of the artificial intelligence (AI) models and the second human grader evaluated on the test set for (A) colour fundus (CF) and (B) fluorescein angiography (FA)

	Accuracy	Precision	Recall	F1-score	AUC-ROC	AUC-PRC
**(A) CF**						
Contrast*						
Manual	0.777	0.553	0.938	0.696	0.828	0.791
DL	0.852	0.653	0.977	0.783	0.956	0.903
Focus*						
Manual	0.816	0.660	0.982	0.789	0.852	0.849
DL	0.905	0.794	0.986	0.880	0.974	0.959
Illumination						
Manual	0.847	0.954	0.367	0.530	0.684	0.748
DL	0.656	0.400	0.921	0.558	0.865	0.737
Shadow and reflection						
Manual	0.705	0.490	0.940	0.644	0.768	0.732
DL	0.731	0.517	0.806	0.630	0.854	0.751
Overall quality						
Manual	0.904	0.849	0.958	0.900	0.912	0.928
Deep Learning AI model	0.919	0.937	0.879	0.907	0.963	0.966
Average*						
Manual	0.771	0.590	0.946	0.717	0.819	0.796
Deep Learning AI model	0.813	0.660	0.914	0.751	0.922	0.863
**(B) FA**						
Contrast*						
Manual	0.651	0.429	0.973	0.596	0.745	0.709
Deep Learning AI model	0.775	0.549	0.840	0.664	0.882	0.717
Focus*						
Manual	0.672	0.531	0.917	0.673	0.718	0.748
Deep Learning AI model	0.818	0.747	0.762	0.755	0.880	0.802
Noise						
Manual	0.773	0.430	0.846	0.570	0.803	0.670
Deep Learning AI model	0.700	0.354	0.839	0.498	0.873	0.722
Overall quality*						
Manual	0.780	0.664	1.000	0.798	0.795	0.839
Deep Learning AI model	0.830	0.755	0.903	0.822	0.918	0.889
Average*						
Manual	0.719	0.513	0.934	0.659	0.765	0.742
Deep Learning AI model	0.781	0.601	0.836	0.685	0.888	0.782

Accuracy, precision, recall, F1-score, AUC-ROC and AUC-PRC have been calculated for each category. In addition, the average over all categories is provided. Statistical significant differences between the AI model and human grader results are indicated with an asterix.

AUC-PRC, area under the precision recall curve; AUC-ROC, area under the receiving operator characteristic curve.

For external evaluation on the EyeQ test set, the provided labels from 0 to 2 were adapted in three ways to match our binary predictions on overall quality. First, intermediate ‘Usable’ quality samples were dropped achieving an accuracy/precision/recall/F1-score/AUC-ROC/AUC-PRC of 0.91/0.81/0.85/0.83/0.95/0.93, comparable to the performance reported by Fu *et al*.^6^ When interpreting intermediate samples as poor quality, the model achieves an accuracy/precision/recall/F1-score/AUC-ROC/AUC-PRC of 0.82/0.53/0.85/0.65/0.91/0.79, and 0.76/0.86/0.61/0.71/0.83/0.85 for intermediate labels being interpreted as good quality.

**Figure 2:**
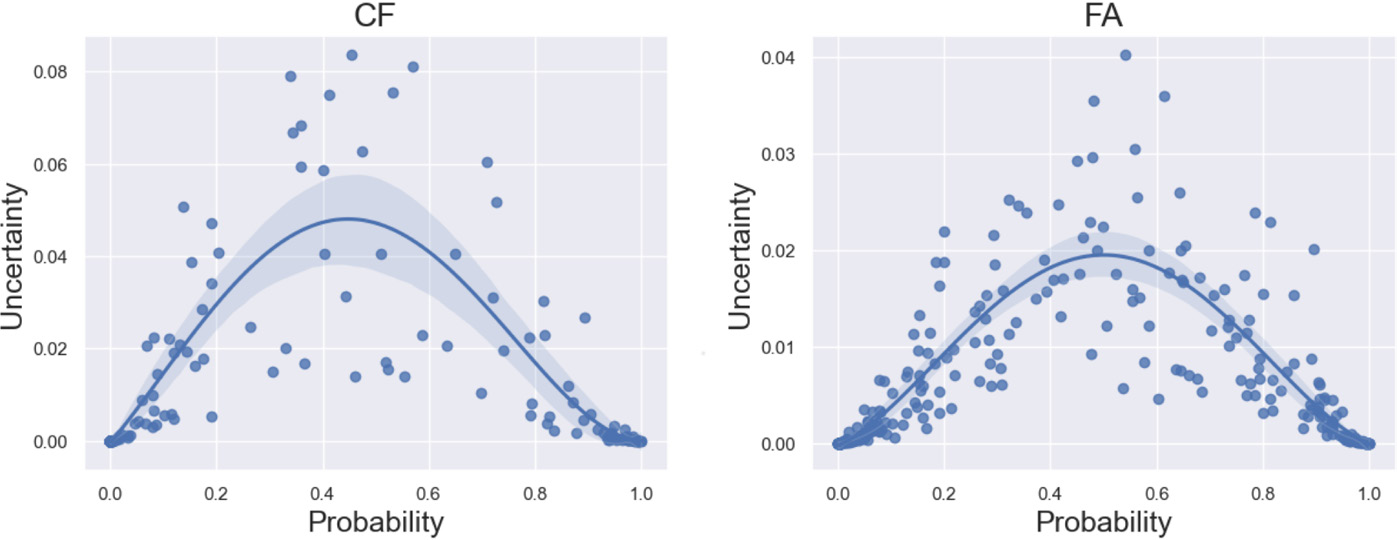
Visualisation of the correlation of the model uncertainty with the predicted probability of the overall quality category, of the ‘CF quality’ (left) and ‘FA quality’ (right) test sets. Each dot represents the ‘overall quality’ prediction for a single sample. While the Y-axis represents the uncertainty score, the X-axis indicates the predicted probability score.

Furthermore, the uncertainty provided by the AI models is related with the classification performance. Comparing the mean/median uncertainty of correctly versus incorrectly predicted samples across all categories, the uncertainty score increases from 0.010/0.0005 to 0.23/0.014 for CF and from 0.006/0.001 to 0.014/0.01 for FA. We also conducted an experiment where we excluded samples with highest uncertainty estimation from the evaluation: after excluding 10%/20%/30% of all samples in the test set with highest uncertainty, total accuracy increases in both datasets: 0.83/0.84/0.87 compared with 0.81 for CF and 0.83/0.86/0.88 compared with 0.78 for FA. Additionally, the uncertainty also correlates with the predicted quality score. The uncertainty is lower for predictions which are either close to 0 (best quality) or 1 (worst quality), and higher for predictions in between (figure 2), having a Pearson correlation coefficient of −0.672 for CF and −0.684 for FA indicating moderate correlation.^11^

### Qualitative results

**Figure 3:**
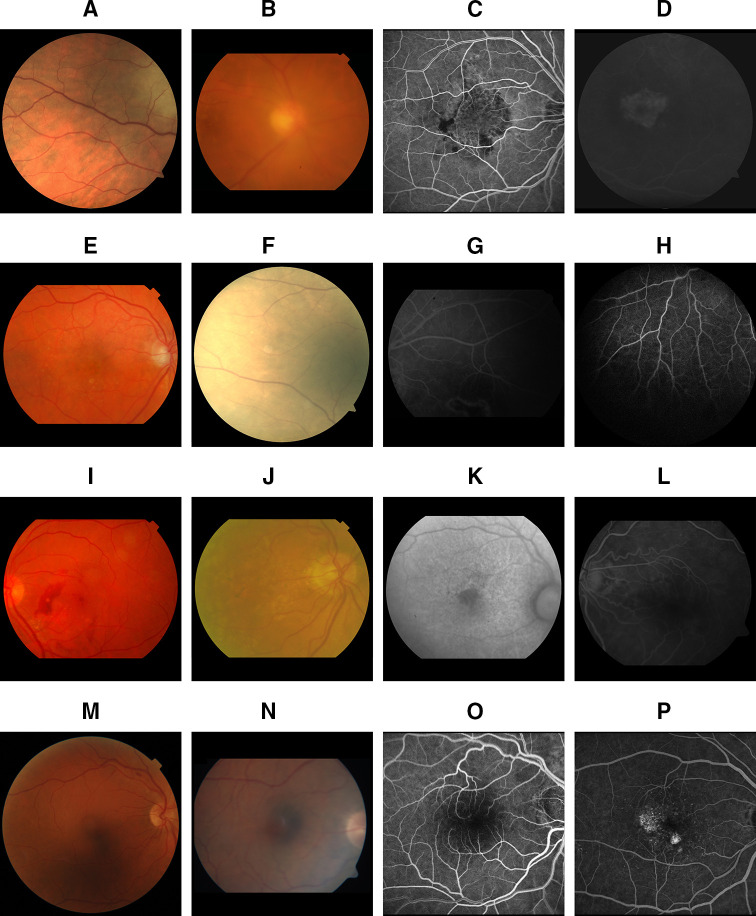
The first row (A–D) shows clear cases with correct predictions and low uncertainty predicted by the network. The sample scans (A) (colour fundus (CF)) and (C) (fluorescein angiography (FA)) are both images with very good quality. (B) A CF example of severe poor quality in focus, where the blurry characteristic makes it hard to see small details (eg, distinguishing small vessels from the background). (D) Poor quality in contrast due to the early phase in FA image acquisition, where no contrast fluid has entered the vessels yet. The second row (E–H) illustrates four examples of border cases with correct predictions of all quality categories. In contrast to the samples above, these scans have been labelled with intermediate quality by human graders. The scans in (I–L) show border cases with high uncertainty and incorrect predictions by the neural network in one or more categories. For all four images, the human grader has assessed the image with intermediate quality (quality score of 2 or 3) in multiple categories. While the manual annotation does not deviate much from the predictions of the AI model, it leads to misclassification when binarised in at least one of the categories. (M–P) Samples with incorrect predictions with comprehensible mistakes by the AI model. (M) A CF image with a shadow artefact on the lower half of the scan, causing a prediction for poor illumination. In (N), the combination of the dark macula at the edge of the CF scan and the bright optic nerve head lead to a prediction of shadow and reflection artefacts. (O) An example for device-dependent noise. While this level of noise is poor quality for images acquired with one device, it could be of good quality for another. (P) An FA image with disease-related artefacts visible as bright spots which have been misinterpreted by the network as noise.

Representative qualitative results were manually selected for distinct cases with correct predictions, borderline cases and comprehensible mistakes by the model for both CF and FA (figure 3).

### Clinical trial use-case evaluation

**Figure 4:**
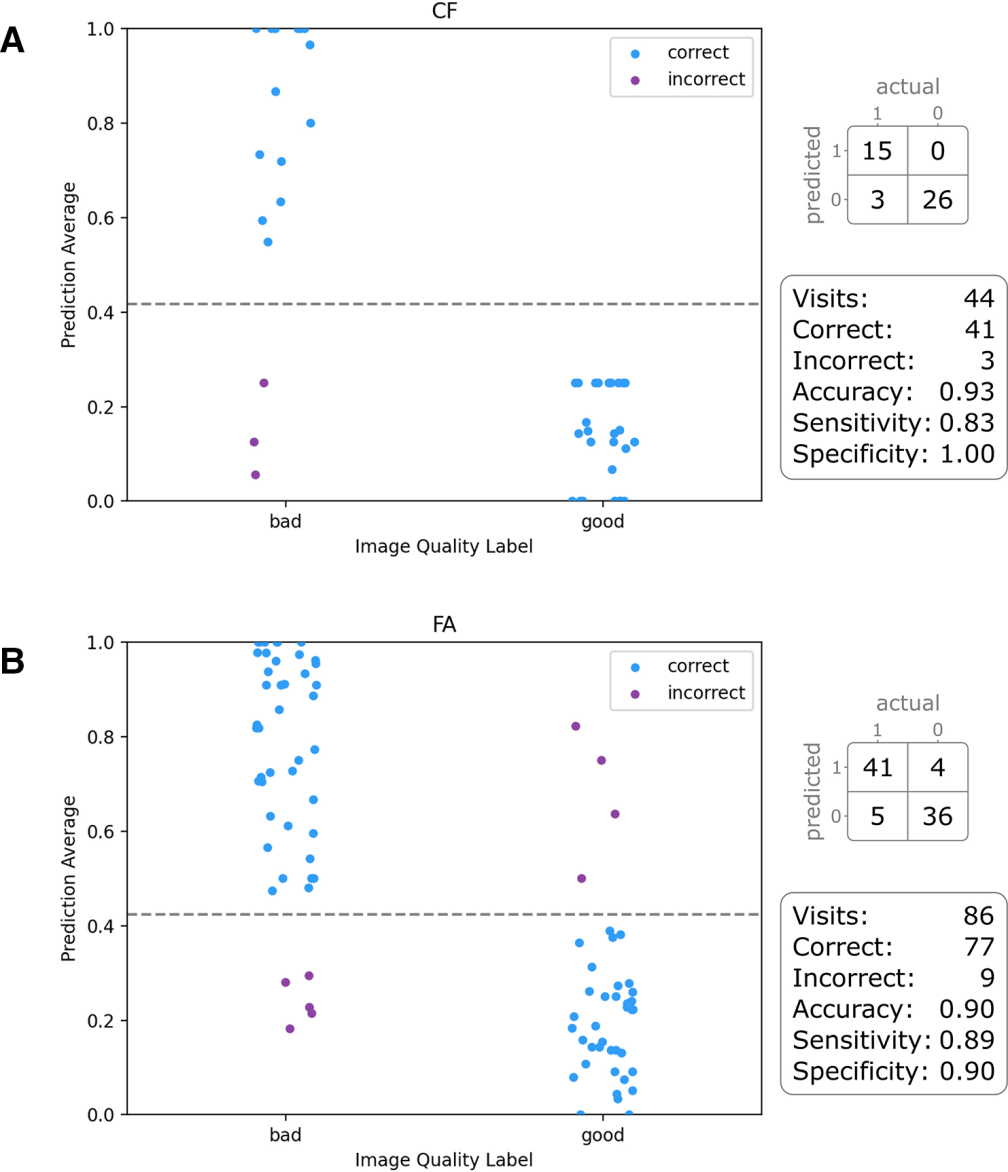
Scatterplots showing the results of the clinical trial use-case evaluation on the test set for (A) colour fundus (CF) and (B) fluorescein angiography (FA), each dot representing a visit. While the X-axis denotes the manual annotation, the prediction probability of the artificial intelligence model for good image quality is plotted on the Y-axis.

Prediction of a full visit takes between 3 and 9 s, depending on the number of images within a visit. For CF the AI model predicted 41 out of 44 visits correctly compared with the manual annotations, misclassifying only three samples, resulting in an accuracy of 0.930. For FA, we evaluated the model on a set of 86 visits, achieving an accuracy of 0.895 with only 9 samples misclassified. The results are visualised in figure 4.

## Discussion

We propose a deep learning approach to automatically predict image quality scores for CF and FA images, achieving human-level performance in our test set and high accuracy in our clinical trial use-case evaluation. To automatise this task of quality assessment for CF and FA, the models produce predictions for multiple image quality categories in a fast and upscale-able way.

Images play an increasingly important role in ophthalmology since the invention of the fundus camera in 1910, especially due to the increasing possibilities of higher resolution leading to unprecedented real-life documentation. Achieving good quality images is crucial in this context to correctly identify diseases and treat patients accordingly, making IQA a significant aspect of daily clinical practice. However, due to the increasing amount of data, IQA poses a resource-intensive step which is not manually applicable in most scenarios. Automatisation of IQA can drastically reduce required resources, save valuable time through real-time feedback on acquired images and help making IQA applicable in clinical practice or clinical trials. Moreover, automated IQA also represents a crucial preprocessing step to make deep learning pipelines more robust and avoid inaccurate predictions of subsequent models.

Automated IQA as proposed in this work is able to give feedback on multiple image quality categories simultaneously in real time, allowing the operators of the imaging console to reacquire images immediately and react to specific poor quality, for example, by adjusting parameters like refractive error of the eye, using lubricating/mydriatic eye drops or preventing movements causing artefacts. Even for FA as an invasive modality, where reacquisition is not possible right away, clinicians can react during the FA imaging process by responding to bad quality assessment on the spot. Particularly in multicentre study settings, real-time IQA can drastically reduce patient burden, avoiding the need for reacquisition of images within a new visit, for example, if images are not sufficient for evaluation at a later point in time by central reading centres. Moreover, automated IQA also helps to overcome the problem of human intergrader/intragrader variability by providing objective and reproducible results. Again, this is of particular relevance in multicentre settings where image evaluation needs to be harmonised across study sites.

Automatisation of IQA is an active field of research in ophthalmology.^1–6 10 12–21^ On one hand, conventional machine learning approaches^10 14–16^ use hand-crafted features, limiting their performance and application domains. On the other hand, most existing deep learning approaches^13^ only classify the overall quality into good or poor^1 17 18^ or introduce a third, intermediate quality.^1 6 19 21^ While a few methods predict the quality in specific categories,^1 3^ they only return the most prominent poor image quality class. In the IEEE ISBI 2020 challenge 5,^22^ labels in the fundus specific categories ‘Artifact’, ‘Clarity’, ‘Field definition’ as well as ‘Overall Quality’ are suggested. In contrast, we propose predictions for multiple more general image quality categories. This represents a new level of detail and improves IQA interpretability for human operators in terms of explainable AI.

In addition, the developed network provides an uncertainty estimate per predicted quality score. Results show that prediction accuracy and uncertainty are related, demonstrating that the calculated uncertainty is a relevant metric for model decisions and can be used as an indicator for requesting human verification. Furthermore, the quality score and uncertainty showed a moderate correlation, mimicking behaviour of human operators and therefore following expected decision patterns. We are convinced that adding these layers of transparency for predictions will help integrating automated approaches into clinical routine, since cases with high uncertainty can be filtered and reviewed by clinicians. The utility of this indicator could be further improved by using the validation set to calibrate the model output, enabling binary indication of human revision at a certain level of uncertainty. However, this is beyond the scope of this study and is left to future work.

In the external validation dataset for CF, the presented model achieved comparable results to the state-of-the-art method trained on the external dataset^6^ when evaluating on two quality classes. When integrating the third ‘Usable’ quality into evaluation, the performance of our proposed model drops, but still achieves reasonable results considering the imprecision introduced with the 3 to 2 class transformation.

Furthermore, the proposed DL approach achieves results on par with or better than the second human grader. We hypothesise that this is partly caused by the subjectiveness in perception of image quality. Both the manual grading as well as the developed networks achieve the best performance in the overall quality category (table 1, online supplemental file 1). We hypothesise that an overall quality prediction naturally clusters images into ‘good’ and ‘any poor’ quality, depicting a significantly easier task than the recognition of specific poor image quality characteristics. In contrast, poor quality images of other categories might resemble each other and cause a higher variability within annotations, as validated through the performance of the second manual test set grading.

When analysing the qualitative examples, wrong predictions are of particular interest (figure 3). We hypothesise that border cases depend on network thresholds and may be improved through additional training data. Another challenge are incorrect predictions through confusion of similar categories. For instance, shadow artefacts/poor illumination have similar appearance in form of a dark segments covering parts/the whole scan. This misclassification would have severe impact on further actions in clinical practice: Depending on the shadowing structure nothing may be changed by the photographer, whereas in case of illumination problems, a pupil dilation or better centralisation of the light into the eye can significantly improve image quality. Future work should aim for improving automated IQA for these cases. Another known problem is that devices of various manufacturers differ in achieved image quality due to used hard- and software.^23 24^ While a specific level of noise would be considered as good quality for images acquired with one device, it may be poor quality when taken with another device. One possible way to tackle this problem is to create separate networks, one per device, which however at the same time amplifies other problems like data scarcity. Landmarks of the eye with unusual appearance or lesions may also be misclassified as they may visually look similar to poor quality characteristics, depicting explicitly hard cases for automated IQA. In our study, the relatively small amount of such cases in the training dataset poses a limitation of the presented approach. The performance for all categories may be improved by adding additional samples for training, as CNNs usually perform better when trained on more data.^25^

Nevertheless, in our additional clinical trial use-case evaluation, results demonstrate high accuracy of 0.930 and 0.895 for both modalities on visits of multiple devices from multiple clinical sites, making it promising for future usage. While calculating the mean of the individual predictions seems to be a simple yet effective method to retrieve visit-level scores, it also poses limitations. For instance, images of the peripheral retina might be not as important compared with macula or optic-disk centred images to make a certain diagnosis. This means that more task-specific strategies should be developed in future work.

Another challenge are early phase images in FA which naturally tend to have low contrast and illumination until the fluorescein as contrast agent becomes visible. This means that they are likely to be incorrectly predicted as poor quality. With an increasing number of such early phase images within a visit, the chance of misclassification of the overall visit quality also increases. One possible solution is to incorporate time information into the model, allowing to weight the impact of individual images on the overall visit prediction accordingly.

In conclusion, we propose a deep learning approach for automated quality assessment of retinal CF and FA images. With 3 and 4 modality-specific categories plus an overall quality together with an uncertainty score for these predictions, we introduce a more efficient prediction than existing approaches while achieving human-level results. Furthermore, the approach is extended to also perform a visit level classification, which was successfully validated within our clinical trial use case. With the help of this work, automated IQA can be integrated into the clinical workflow convincingly and advance the process of ophthalmological examinations for more efficient and effective disease management.

## Data Availability

Data are available upon reasonable request. Unfortunately, due to privacy restrictions, the image dataset can not be made publicly available. However, access to the data may be shared upon reasonable request.
