# Correction: *Quality assessment of colour fundus and fluorescein angiography images using deep learning*

**DOI:** 10.1136/bjo-2022-321963corr1

**Published:** 2026-01-14

König M, Seeböck P, Gerendas BS, *et al.* Quality assessment of colour fundus and fluorescein angiography images using deep learning. *Br J Ophthalmol* 2023:18;108:98–104. doi: 10.1136/bjo-2022–3 21 963.

During the production of this paper, the joint first authorship statement was omitted. This has now been updated to the following: ‘Michael König and Philipp Seeböck equally contributed as first authors’.

