# Supplementary material for: Quality assessment of colour fundus and fluorescein angiography images using deep learning
Source: Br J Ophthalmol. 2022 Nov 23;108(1):98–104. doi: 10.1136/bjo-2022-321963 (PMC10804038; doi:10.1136/bjo-2022-321963)
Supplement: Supplementary data [file bjo-2022-321963supp001.pdf]

Supplementary Information for

Quality Assessment of Color Fundus and Fluorescein Angiography Images using Deep Learning

Michael König[1,*], Philipp Seeböck[1,*], Bianca S. Gerendas[1], Georgios Mylonas[1], Rudolf Winklhofer[1], Ioanna Dimakopoulou[1], Ursula Schmidt-Erfurth[1]

Affiliations:

[*] These authors contributed equally.
[1] Department of Ophthalmology and Optometry, Medical University of Vienna, Vienna, Austria.

Table of contents:

- **Supplemental Text:** Method Details, Evaluation Details
- **Supplemental Figures:** eFigure 1, eFigure 2, eFigure 3
- **Supplemental Tables:** eTable 1, eTable 2, eTable 3, eTable 4, eTable 5
- **Supplemental References**

König M, *et al. Br J Ophthalmol* 2023; 108:98–104. doi: 10.1136/bjo-2022-321963

Methods Details

Baseline method The approach of Sadeghipour et al.[1] is used as a baseline approach for automated image quality assessment, allowing to put the results of the proposed deep learning (DL) model in better context. This baseline is built on a handcrafted feature-based machine learning approach by Dias et al.[2], utilizing an ensemble of networks for quality assessment, one per category. For each category, custom hand-crafted features are extracted from the input image and used to train a Support Vector Machine, naive Bayes classifier, classification tree and AdaBoost classifier. The model with the best validation performance is then selected as this category's classifier. Furthermore, a last classifier is trained using the predicted quality scores of all categories as input, predicting an overall quality score.

Pre-processing Images have been resized to 512 x 512 pixel before getting processed by the network. To keep the aspect ratio, the larger side was scaled to 512 pixels, while the smaller side was scaled in correct ratio and padded evenly on both sides with black pixels. Furthermore, different image augmentation techniques have been randomly applied during training. Random flipping in vertical and horizontal direction with a probability of 0.5, random rotation between -15 and +15 degree, vertical/horizontal translation of up to 20%/10% of the image size as well as scaling of ±10% have been applied.

Architecture The architecture of the proposed neural network follows a conventional ResNet18 structure as proposed by He et al.[3]. This network is composed of an initial convolution, followed by 4 ResNet layers, a global average pooling and a fully connected layer followed by a sigmoid function, forming the model output. The initial convolution consists of a convolutional layer followed by

batch normalization layer, rectifying linear unit (ReLu) and max-pooling operation. A ResNet layer is composed of two convolutional blocks, each consisting of a convolutional and batch normalization layer, with an intermediate ReLu between those two blocks. Furthermore, a residual connection is joining information before and after the ResNet layer. However, to be able to apply the principle of Monte Carlo dropout[4] and produce an uncertainty score for predictions, dropout layers have been added after each ResNet Layer of the network. The model concludes with a fully connected layer with 4 or 5 output neurons, depending on the input modality. Each neuron provides a quality score between 0 and 1 for a specific category. A visualization of the architecture is shown in **eFigure 3**.

Training details Training of the DL models was performed using a minibatch size of 32. Both models were trained for 20000 iterations halted by 200 uniformly distributed calculations of the validation performance. A pre-trained ResNet18 with added dropout layers (p=0.2) was trained using the Adam optimizer with standard parameters and a learning rate of $5*10^{-4}$. The binary cross entropy loss was computed for each output category and combined into a final overall loss using the (equally weighted) average:

$$Loss_{BCE} = -\frac{1}{N}\sum_{n=1}^{N} y_n * \log(x_n) + (1 - y_n) * \log(1 - x_n) \qquad (f1)$$

y_n denotes the nth value in the model output, x_n the corresponding ground truth and N the total number of values in the model output.

Image to visit level transformation In clinical daily routine, a whole series of images is acquired during a single patient visit. For CF a visit typically consists of 3 to 8 images per eye, showing different retinal areas according to predefined protocols like the Early Treatment Diabetic Retinopathy Study definition[5]. For FA a visit is

composed of an image series of the retina covering a timespan of up to 20 minutes. Therefore, predictions somehow have to be transformed from image to visit level. To achieve this, we first apply the trained final model to each image of the visit iteratively. Second, the mean of all individual predictions is calculated to combine the individual results. Finally, a binary prediction on the quality of the whole visit is produced by applying a threshold (CF: 0.417, FA: 0.424) to this mean value. The thresholds have been optimized on the validation set to allow an unbiased estimation of the performance on the test set.

Evaluation Details

Dataset Heterogeneity The used dataset consists of images acquired by more than 200 clinical sites, different device manufacturers, varying diseases and pixel resolutions, ranging from 496 x 512 to 6000 x 4000 pixels. Details regarding the distribution of image pixel resolutions in X and Y direction among training, validation and test set for CF and FA are visualized in **eFigure 1**. Information with respect to imaging devices per manufacturer is provided in **eTable 1.** The distribution of images per manufacturer and diseases among datasets and in total for CF and FA is shown in **eTable 2**.

Metrics details Accuracy describes the amount of correct predictions, precision the proportion of positive predictions which are actually positive, recall the fraction of positive predictions out of all positive examples and F1-score the harmonic mean of precision and recall.[6] Both AUC measures provide insight into the model performance among changing thresholds and therefore indicate performance stability.

Test set details Data with regression labels used for evaluating the models was divided into a validation and test set on a patient level with an approximate ratio of 1:2. The validation set was utilized for monitoring network training and threshold calculations, while the test set was used for final performance evaluation. For all 264/321 test set images of CF/FA, the mean of human labels per category are shown in **eTable 4** before and after binary transformation. The test sets of the clinical trial use-case were constructed as a balanced dataset of 44 (CF) and 86 (FA) visits, with a 50% share of good and bad quality samples each. After processing of the dataset

through the model, ground truth was revised and lead to a share of good quality visits of 0.59 (CF) and 0.47 (FA).

Likert scale evaluation The model was trained on binary labels. To enable more detailed evaluation of prediction error, specially for borderline cases, evaluation samples were annotated with labels of higher granularity. In this experiment, the distribution of good/poor quality predictions provided by the model per Likert scale ground truth label are visualized (**eFigure 2**). For CF a clear trend of increasing number of positive predictions with increasing Likert scale label is visible, with the most even distribution of positive and negative predictions for label 3. For FA, a similar trend is visible apart from a deviation from label 4 to 5.

Image size Quality is assessed in multiple general image quality categories, which might consist of artifacts sensitive to the size of images (e.g. focus, noise). We therefore conducted an experiment to evaluate the impact of input image sizes on model performance. Four models were trained per modality (CF, FA) on the same training images with different rescaling factors, resulting in image pixel sizes of 256 x 256, 512 x 512, 1024 x 1024 and 1532 x 1532. Average results do not show any significant differences for the evaluated metrics in both modalities (**eTable 3a, eTable 3b**). Exemplary, the performance of all four models is provided for the category ‘noise’ in **eTable 3c**, showing a certain relation between image resolution and model performance. While accuracy, precision and F1-Score improve with increasing image size, recall, and both area under the curve metrics (AUC-ROC, AUC-PRC) achieve best results for an image size of 512 x 512 pixels.

Comparison of traditional machine learning baseline and proposed approach In **eTable 5** quantitative results for the hand-crafted feature baseline[1],

the proposed deep learning approach and the second human grader are presented. Our method clearly outperforms the baseline, showing higher numbers in almost all metrics in both modalities. While critical performance drops can be seen in multiple categories for the baseline approach, the proposed model shows a more stable behavior.

These results are in line with findings on convolutional neural networks (CNN) based approaches outperforming conventional feature based methods[7]. However, when considering accuracy and precision, the baseline seems to occasionally achieve better results than the presented approach. At the same time, the used dataset is not balanced for each category (**eTable 4**), meaning that accuracy is less conclusive compared to other measures (e.g. classification of all samples as good quality could result in high accuracy due to the lower amount of bad quality samples). Furthermore, precision and recall are interdependent metrics and should be considered only together, e.g. in form of their harmonic mean (F1-score).

König M, *et al. Br J Ophthalmol* 2023; 108:98–104. doi: 10.1136/bjo-2022-321963

Supplemental Figures

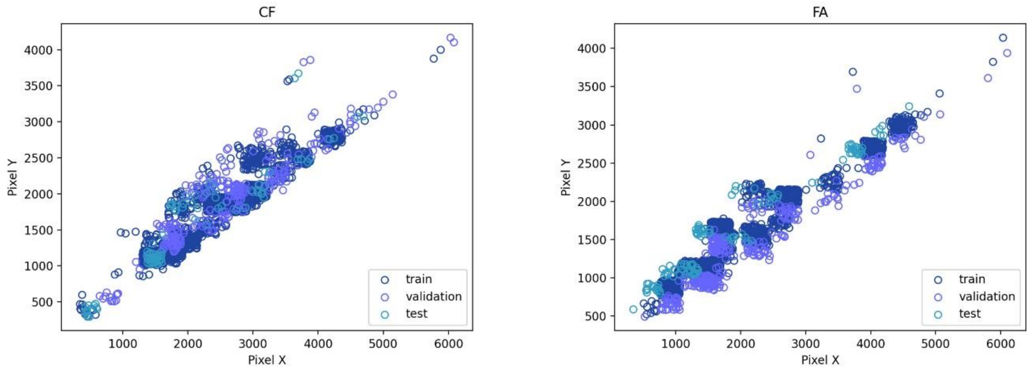

eFigure 1: Resolution of images in X and Y direction among training, validation and test set for CF (left) and FA (right). A small jitter has been added to better visualize overlapping samples.

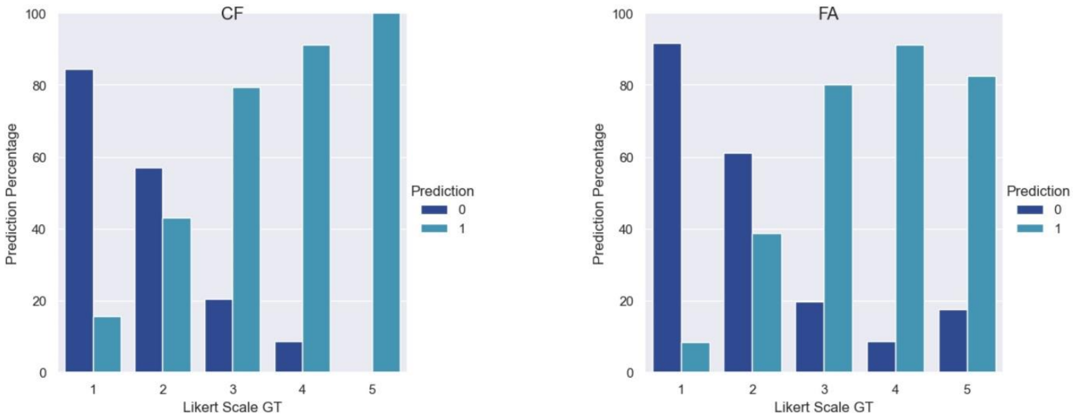

eFigure 2: Percentage of good (0) and poor (1) quality model predictions (Y-axis) per Likert scale ground truth label (X-axis) for CF (left) and FA (right).

König M, *et al. Br J Ophthalmol* 2023; 108:98–104. doi: 10.1136/bjo-2022-321963

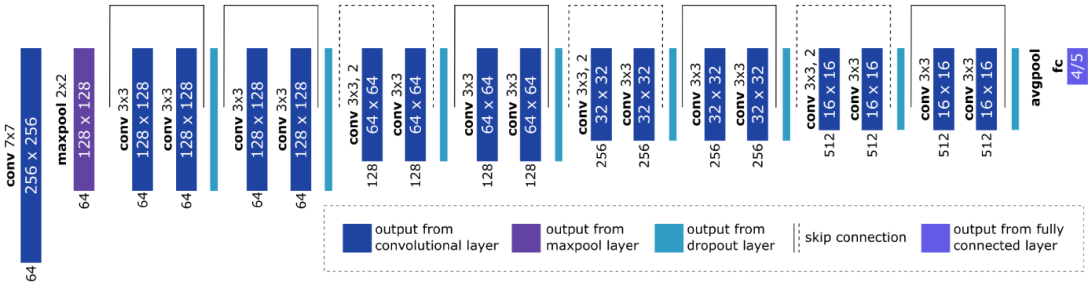

eFigure 3: Architecture of the proposed model. While following a conventional ResNet18 structure, a dropout layer has been added after each ResNet layer. Skip-connections are depicted both as solid and dotted lines, where solid lines represent connections with a constant number of channels and dotted lines implicate a connection where the number of channels increase while the spatial dimensions decrease. The final layer is comprised of four output neurons for FA and five for CF.

Supplemental Tables

eTable 1: List of imaging devices per manufacturer included in the provided datasets.

Manufacturer	*Devices*
Canon	CF-60DSi, CF-60UV, CF-60UVi, CF-1, CR-1, CR-2, CR-DGI, CX-1, EOS 5D, EOS 40D
Clarity	RetCam 2, RetCam 3, RetCam Shuttle
Heidelberg	Spectralis HRA+OCT
Kowa	KD-211C, Nonmyd α-DIII, VX-10α, VX-10i
Nidek	AFC-210, AFC-330
Topcon	Topcon 1000, Topcon 2000, 2000 FA Plus, DRI Triton, TRC-50DX, TRC-50EX,<br>TRC-50IX, TRC-NW6S, TRC-NW7SF, TRC-NW8, TRC-NW400
Zeiss	FF4, FF450+, Visucam 500, Visucam 524, Visucam NM/FA, Visucam Pro NM

König M, *et al. Br J Ophthalmol* 2023; 108:98–104. doi: 10.1136/bjo-2022-321963

eTable 2: Distribution of images per manufacturer and diseases among datasets and in total for (a) CF and (b) FA.

(a) CF

Manufacturer	*Train*	*Validation*	*Test*	*Total*
Canon	0.165	0.046	0.130	0.157
Clarity	0.036	0.471	0.156	0.066
Kowa	0.074	0.023	0.061	0.071
Nidek	0.002	0.023	0.016	0.004
Topcon	0.322	0.276	0.304	0.318
Zeiss	0.400	0.161	0.335	0.384

Disease				
DME	0.769	0.345	0.418	0.712
Macular Edema	0.099	0.115	0.008	0.089
Neovascular AMD	0.100	0.437	0.410	0.149
Retinopathy of Prematurity	0.032	0.103	0.156	0.049

(b) FA

Manufacturer	*Train*	*Validation*	*Test*	*Total*
Canon	0.108	0.119	0.080	0.105
Heidelberg	0.332	0.331	0.288	0.326
Kowa	0.045	0	0.025	0.040
Topcon	0.160	0.339	0.347	0.192
Zeiss	0.356	0.212	0.260	0.337

Disease				
AMD	-	0.017	0.003	0.001
DME	0.138	0.237	0.288	0.162
Macular Edema	0.206	0.203	0.161	0.200
Neovascular AMD	0.656	0.533	0.539	0.635
Vitreomacular Traction	-	0.008	0.009	0.002

König M, *et al. Br J Ophthalmol* 2023; 108:98–104. doi: 10.1136/bjo-2022-321963

eTable 3: Performance (accuracy, precision, recall, F1-score, AUC-ROC, AUC-PRC) of four models trained and evaluated on different image sizes (256, 512, 1024, 1532) on the test set. While (a) the table on the top shows the performance results averaged over all target categories of CF, (b) the middle table provides the average performance results for FA. As an exemplary result, (c) the table on the bottom illustrates metrics for the modality specific category 'noise'.

(a) CF averaged

Image Size	*Accuracy*	*Precision*	*Recall*	*F1-score*	*AUC-ROC*	*AUC-PRC*
256	0,811	0,670	0,890	0,750	0,928	0,874
512	0,791	0,633	0,927	0,736	0,923	0,859
1024	0,833	0,689	0,863	0,758	0,916	0,857
1532	0,806	0,651	0,883	0,740	0,921	0,846

(b) FA averaged

Image Size	*Accuracy*	*Precision*	*Recall*	*F1-score*	*AUC-ROC*	*AUC-PRC*
256	0,775	0,606	0,755	0,659	0,863	0,773
512	0,793	0,610	0,833	0,695	0,887	0,783
1024	0,782	0,604	0,825	0,685	0,889	0,811
1532	0,823	0,677	0,709	0,693	0,870	0,786

(c) FA Noise

Image Size	*Accuracy*	*Precision*	*Recall*	*F1-score*	*AUC-ROC*	*AUC-PRC*
256	0,700	0,332	0,682	0,446	0,767	0,632
512	0,757	0,407	0,809	0,541	0,870	0,719
1024	0,802	0,460	0,689	0,552	0,838	0,700
1532	0,839	0,542	0,582	0,561	0,817	0,695

König M, *et al. Br J Ophthalmol* 2023; 108:98–104. doi: 10.1136/bjo-2022-321963

eTable 4: Mean of human labels of used test sets for (a) CF and (b) FA before and after transformation of regression labels into binary labels.

(a) CF	*Contrast*	*Focus*	*Illumination*	*Shadow & Reflection*	*Overall Quality*	*Average*
Regression	2.138	2.458	2.054	2.055	2.686	2.278
Binary	0.333	0.408	0.258	0.303	0.502	0.361

(b) FA	*Contrast*	*Focus*	*Noise*	*Overall Quality*	*Average*
Regression	2.038	2.243	1.844	2.558	2.171
Binary	0.277	0.385	0.196	0.464	0.330

König M, *et al. Br J Ophthalmol* 2023; 108:98–104. doi: 10.1136/bjo-2022-321963

eTable 5: Quantitative results of the baseline method, the second manual grading and the proposed DL approach on the test set for (a) CF and (b) FA. Accuracy, precision, recall, F1-score, AUC-ROC and AUC-PRC have been calculated for each category. In addition, the average across all categories is provided.

(a) CF		*Accuracy*	*Precision*	*Recall*	*F1-score*	*AUC-ROC*	*AUC-PRC*
Contrast	baseline	0.858	0.823	0.610	0.700	0.802	0.817
	manual	0.777	0.553	0.938	0.696	0.828	0.791
	DL	0.852	0.653	0.977	0.783	0.956	0.903
Focus	baseline	0.761	0.613	0.871	0.719	0.796	0.804
	manual	0.816	0.660	0.982	0.789	0.852	0.849
	DL	0.905	0.794	0.986	0.880	0.974	0.959
Illumination	baseline	0.654	0.398	0.911	0.554	0.735	0.679
	manual	0.847	0.954	0.367	0.530	0.684	0.748
	DL	0.656	0.400	0.921	0.558	0.865	0.737
Shadow & Reflection	baseline	0.710	0.491	0.666	0.566	0.696	0.640
	manual	0.705	0.490	0.940	0.644	0.768	0.732
	DL	0.731	0.517	0.806	0.630	0.854	0.751
Overall Quality	baseline	0.353	0.264	0.241	0.252	0.345	0.469
	manual	0.904	0.849	0.958	0.900	0.912	0.928
	DL	0.919	0.937	0.879	0.907	0.963	0.966
Average	*baseline*	*0.706*	*0.629*	*0.551*	*0.553*	*0.664*	*0.696*
	manual	*0.771*	*0.590*	*0.946*	*0.717*	*0.819*	*0.796*
	DL	*0.813*	*0.660*	*0.914*	*0.751*	*0.922*	*0.863*

(b) FA		*Accuracy*	*Precision*	*Recall*	*F1-score*	*AUC-ROC*	*AUC-PRC*
Contrast	baseline	0.808	0.654	0.580	0.615	0.737	0.681
	manual	0.651	0.429	0.973	0.596	0.745	0.709
	DL	0.775	0.549	0.840	0.664	0.882	0.717
Focus	baseline	0.573	0.453	0.786	0.575	0.620	0.672
	manual	0.672	0.531	0.917	0.673	0.718	0.748
	DL	0.818	0.747	0.762	0.755	0.880	0.802
Noise	baseline	0.823	0	0	0	0.500	0.598
	manual	0.773	0.430	0.846	0.570	0.803	0.670
	DL	0.700	0.354	0.839	0.498	0.873	0.722
Overall Quality	baseline	0.793	0.789	0.716	0.751	0.779	0.822
	manual	0.780	0.664	1.000	0.798	0.795	0.839
	DL	0.830	0.755	0.903	0.822	0.918	0.889
Average	*baseline*	*0.749*	*0.632*	*0.521*	*0.485*	*0.659*	*0.693*
	manual	*0.719*	*0.513*	*0.934*	*0.659*	*0.765*	*0.742*
	DL	*0.781*	*0.601*	*0.836*	*0.685*	*0.888*	*0.782*

König M, *et al. Br J Ophthalmol* 2023; 108:98–104. doi: 10.1136/bjo-2022-321963

Supplemental References

1. Sadeghipour A, Arikan M, Ismail O, König M, Baltali B, Gerendas BS, et al. Imageability and Registration of Multimodal Imaging using Machine Learning. Invest Ophthalmol Vis Sci. 2019;60(9):2197.

2. Dias JMP, Oliveira CM, da Silva Cruz LA. Retinal image quality assessment using generic image quality indicators. Information Fusion. 2014;19:73–90.

3. He K, Zhang X, Ren S, Sun J. Deep Residual Learning for Image Recognition. In: Proceedings of the IEEE conference on computer vision and pattern recognition. 2016. p. 770–8.

4. Gal Y, Ghahramani Z. Dropout as a Bayesian Approximation: Representing Model Uncertainty in Deep Learning. In: International Conference on Machine Learning (ICML). 2016. p. 1050–9.

5. Early Treatment Diabetic Retinopathy Study Research Group. Grading Diabetic Retinopathy from Stereoscopic Color Fundus Photographs - An Extension of the Modified Airlie House Classification: ETDRS Report Number 10. Ophthalmology. 1991;98(5):786–806.

6. Philipp Seeböck. Discovery of Biomarker Candidates in Retinal OCT Images using Deep Learning [Internet]. [Vienna]: Medical University of Vienna; 2019 [cited 2022 Apr 20]. Available from: https://optima.meduniwien.ac.at/team/computational-imaging-research/philipp-seebock/

7. Raj A, Tiwari AK, Martini MG. Fundus image quality assessment: survey, challenges, and future scope. IET Image Process. 2019;13(8):1211–24.

König M, *et al. Br J Ophthalmol* 2023; 108:98–104. doi: 10.1136/bjo-2022-321963
